# The Development of an Automated Device for Asthma Monitoring for Adolescents: Methodologic Approach and User Acceptability

**DOI:** 10.2196/mhealth.3118

**Published:** 2014-06-19

**Authors:** Hyekyun Rhee, Sarah Miner, Mark Sterling, Jill S Halterman, Eileen Fairbanks

**Affiliations:** ^1^University of Rochester Medical CenterSchool of NursingUniversity of RochesterRochester, NYUnited States; ^2^Rochester Institute of TechnologyDepartment of Biomedical EngineeringRochester, NYUnited States; ^3^University of Rochester Medical CenterDepartment of PediatricsUniversity of RochesterRochester, NYUnited States

**Keywords:** asthma, adolescents, symptom monitoring, symptom algorithm, mobile device

## Abstract

**Background:**

Many adolescents suffer serious asthma related morbidity that can be prevented by adequate self-management of the disease. The accurate symptom monitoring by patients is the most fundamental antecedent to effective asthma management. Nonetheless, the adequacy and effectiveness of current methods of symptom self-monitoring have been challenged due to the individuals’ fallible symptom perception, poor adherence, and inadequate technique. Recognition of these limitations led to the development of an innovative device that can facilitate continuous and accurate monitoring of asthma symptoms with minimal disruption of daily routines, thus increasing acceptability to adolescents.

**Objective:**

The objectives of this study were to: (1) describe the development of a novel symptom monitoring device for teenagers (teens), and (2) assess their perspectives on the usability and acceptability of the device.

**Methods:**

Adolescents (13-17 years old) with and without asthma participated in the evolution of an automated device for asthma monitoring (ADAM), which comprised three phases, including development (Phase 1, n=37), validation/user acceptability (Phase 2, n=84), and post hoc validation (Phase 3, n=10). In Phase 1, symptom algorithms were identified based on the acoustic analysis of raw symptom sounds and programmed into a popular mobile system, the iPod. Phase 2 involved a 7 day trial of ADAM in vivo, and the evaluation of user acceptance using an acceptance survey and individual interviews. ADAM was further modified and enhanced in Phase 3.

**Results:**

Through ADAM, incoming audio data were digitized and processed in two steps involving the extraction of a sequence of descriptive feature vectors, and the processing of these sequences by a hidden Markov model-based Viterbi decoder to differentiate symptom sounds from background noise. The number and times of detected symptoms were stored and displayed in the device. The sensitivity (true positive) of the updated cough algorithm was 70% (21/30), and, on average, 2 coughs per hour were identified as false positive. ADAM also kept track of the their activity level throughout the day using the mobile system’s built in accelerometer function. Overall, the device was well received by participants who perceived it as attractive, convenient, and helpful. The participants recognized the potential benefits of the device in asthma care, and were eager to use it for their asthma management.

**Conclusions:**

ADAM can potentially automate daily symptom monitoring with minimal intrusiveness and maximal objectivity. The users’ acceptance of the device based on its recognized convenience, user-friendliness, and usefulness in increasing symptom awareness underscores ADAM’s potential to overcome the issues of symptom monitoring including poor adherence, inadequate technique, and poor symptom perception in adolescents. Further refinement of the algorithm is warranted to improve the accuracy of the device. Future study is also needed to assess the efficacy of the device in promoting self-management and asthma outcomes.

## Introduction

### Asthma and Adolescents in the United States

Asthma represents a serious health condition in children and adolescents in the United States, with increasing mortality and morbidity over the past two decades [1]. According to recent national statistics [2], current asthma was reported by over 7 million children (9.6%) ages 17 and younger in the United States, of which, 39% (2.8 million) were adolescents (12-17 years old). Nearly 12% of high school students in the United States reported a current asthma diagnosis in 2011 [3]. Adolescents suffer greater asthma-related morbidity than other age groups [4,5]. Serious adverse outcomes requiring hospitalization, intubations, and cardiopulmonary resuscitation are more common in adolescents than in younger children [6]. Moreover, asthma mortality among adolescents is approximately twice that of younger children [7]. Given the high prevalence and substantial adverse outcomes of asthma and its overall impact on quality of life in adolescents, it is imperative to implement effective strategies that can improve self-management and health outcomes in this population.

### Asthma Self-Management

Prior research has generated compelling evidence that programs promoting self-management can reduce morbidity and improve asthma outcomes in children [8-11]. Successful asthma management strategies require the patients’ active commitment to engage in care processes by establishing self-monitoring routines [12]. Adequate self-monitoring of asthma symptoms is considered to be the cornerstone of appropriate asthma management, leading to fewer cases of asthma exacerbation and acute care visits, as well as better functional outcomes and higher quality of life in children and adolescents [13]. Symptom monitoring informs patient decisions to initiate necessary self-management behaviors (eg, adjust medication, alter activity level, alter the surrounding environment, or seek medical assistance), as well as the health care providers’ decisions related to an appropriate treatment course. Thus, current guidelines by the National Heart, Lung, and Blood Institute Expert Panel Review 3 [14] highlight the importance of ongoing symptom monitoring. Programs that increased patient understanding and perception of asthma control could improve asthma control, quality of life, and reduce acute health care utilization [15].

Nonetheless, studies of children have raised concerns about the adequacy and effectiveness of current methods of asthma self-monitoring, including symptom-based and peak expiratory flow (PEF) monitoring [13,16,17]. Poor adherence and inadequate technique by children further diminish the clinical usefulness of PEF monitoring. The uncertainty of current monitoring strategies underscores the imperative of an alternative symptom monitoring strategy that addresses the issues of accuracy and patient adherence.

Accurate symptom monitoring by patients is the most fundamental antecedent to effective asthma management, yet existing monitoring strategies have not been conducive to adolescents’ cooperation, nor yielded accurate or clinically useful information [13,18-20]. Having recognized these limitations, we developed an innovative device that can facilitate continuous and accurate monitoring of asthma symptoms with minimal disruption of daily routines, thus increasing acceptability to adolescents. The objectives of this paper were to: (1) describe the development of this novel symptom monitoring device to support optimal asthma self-management for teenagers (teens), and (2) assess their perspectives on the usability and acceptability of the device.

## Methods

### Description of an Automated Device for Asthma Monitoring

**Figure 1:**
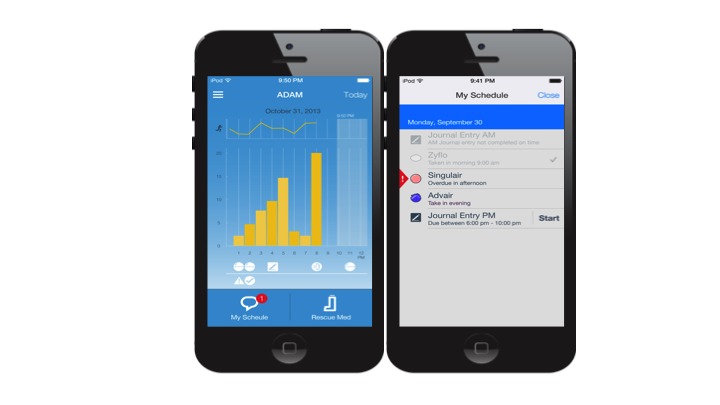
Selected screenshots of the automated device for asthma monitoring (ADAM).

The automated device for asthma monitoring (ADAM) is a novel device that quantifies symptoms in numbers, based on predetermined algorithms of symptom sounds including coughs and wheezes. The device uses the iPod as a platform. It automatically processes and analyzes raw symptom sounds, and displays and stores the number of identified symptoms. It simultaneously monitors activity using the iPod’s built in accelerometer, which allows patients to view the symptoms in relation to their level of activity. Secondary functions of the device include electronic asthma diary keeping, and medication tracking and reminding. Figure 1 shows the selected screenshots of the device.

### Overview of the Study Design for the Development and Validation of the Automated Device for Asthma Monitoring

**Figure 2:**
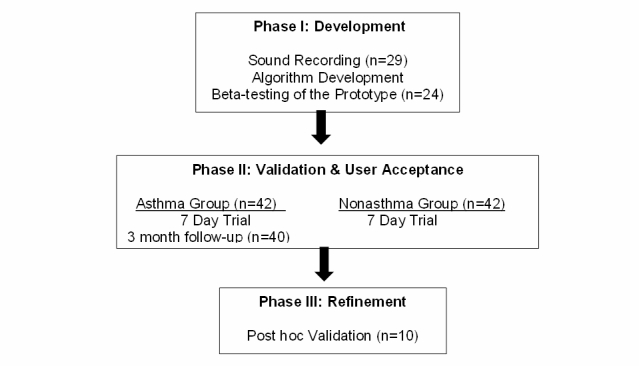
Overview of the study flow from Phase 1 to Phase 3.

This study consisted of three phases, including development (Phase 1), validation (Phase 2), and post hoc validation (Phase 3). During Phase 1, we collected raw symptom sounds that became the basis for the delineating symptom algorithms. A case-control design was used for Phase 2 involving an asthma group and a nonasthma group, who participated in a 7 day trial to determine the validity and feasibility of the new device. In Phase 3, we reevaluated the modified and improved device for its sensitivity and specificity. Figure 2 shows the overall flow of the study phases. Below are the detailed descriptions of the methods implemented in each phase. The Institutional Review Board approved the study protocol for each phase, and informed parental consent and teen assent were obtained for each phase prior to data collection.

### Phase 1: Development Phase

#### Study Design and Sample

**Figure 3:**
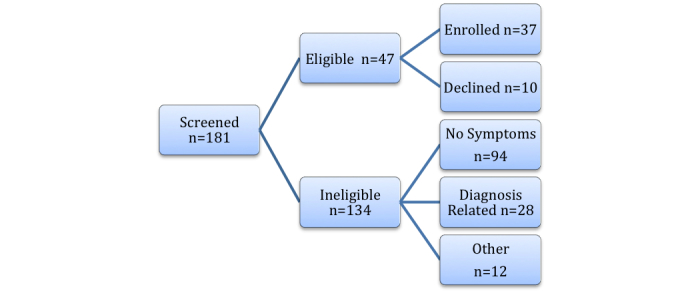
Enrollment flowchart for Phase 1.

To develop symptom algorithms, we collected raw symptom sounds using a digital recorder. The identified algorithms were then programmed in a popular mobile device, the iPod, which was used as a platform to capture, process, and store symptom information. Subsequently, the prototype device was beta-tested with the subjects to evaluate its operation. Subject eligibility criteria were: (1) 13-17 years old, (2) physician-diagnosed asthma for at least one year, (3) active asthma symptoms within the last 24 hours, and (4) ability to understand spoken and written English. Those subjects, with other diagnoses that can produce asthma-like symptoms (eg, cardiac disease, cystic fibrosis) or significant cognitive impairment that would present concerns in following the study protocol, were excluded. There were 29 adolescents that participated in the sound collection stage. Of those adolescents, 16 continued on to participate in the beta-testing period, along with 8 additional subjects who enrolled only for beta-testing. The majority of participants (54%, 20/37) were recruited from the emergency department, and the rest (46%, 17/37) were recruited from pediatric outpatient clinics affiliated with a major academic medical center through clinical referrals or recruitment flyers. Figure 3 shows the numbers of teens from screening to enrollment.

#### Procedure and Instruments

The subjects continuously recorded breathing sounds for 24 hours during the sound collection stage using a digital recorder (Olympus WS-331M). A small external microphone was attached mid line on the shirt collar to amplify the breathing sounds for recording. The subjects carried the recorder either in their clothing pockets or a carrying case. They were instructed to pause the recording during times they desired privacy. Over night, the recorder and microphone were placed on a surface close to the head of the bed to record any nighttime symptoms, while the device was recharging. Each subject provided a minimum of 10 hours of sound recording. The subjects simultaneously completed a 24 hour asthma diary, recording symptoms, feelings, activities, and medication use.

#### Sound Analysis

The recorded sounds were downloaded to a computer, and two research nurses carefully listened independently using audio software, Adobe Audition, to extract the sounds of asthma symptoms including coughing, wheezing, and throat clearing. The sounds of interest were sorted and annotated for further validation and analysis. There were three clinicians, including a pediatrician, a pulmonologist, and the principal investigator (HR) who did the validation. The principal investigator independently evaluated the pool of randomly selected symptom sounds, and classified them into cough, wheeze, and others. They agreed on 96.6% of the cases (116/120 total cases), and any discrepancies that failed to reach agreement were removed from the symptom database.

#### Beta-Testing of the Prototype

Following the initial development of the prototype, a 3 day prototype trial was conducted with 24 teens, of which 16 also participated in the sound recording stage earlier. During the 3 day beta-testing period, the subjects carried the prototype device during the daytime, and kept it running over night while recharging.

### Phase 2: Validation and User Acceptance of the Device

#### Study Design and Sample

**Figure 4:**
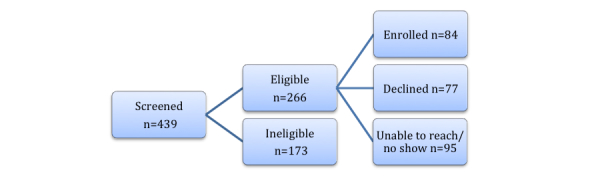
Enrollment flowchart for Phase 2.

This phase used a case-control design involving an asthma group (n=42), and an age matched nonasthma group (n=42) to evaluate the validity of the newly developed device. Figure 4 shows the flow of participants.

The most common reasons for ineligibility were unverifiable current asthma diagnosis for the asthma group, and having past asthma diagnosis or respiratory symptoms for the nonasthma group. Each group participated in a 7 day trial during which the device was used continuously for 24 hours. The asthma group eligibility criteria for Phase 2 were similar to those in Phase 1, except that they were not required to be symptomatic at the time of enrollment. The nonasthma group consisted of those who were with no current/past history of asthma or other health conditions producing asthma-like symptoms, and free of any current respiratory symptoms.

We performed validity testing, comparing results among adolescents with and without asthma, and correlating data from the asthma group to other measures of the asthma condition, including Forced Expiratory Volume in 1 second, Asthma Control Test, **Fractional exhaled nitric oxide**, daily symptom diaries, visual analogue scale, health care utilization, and asthma related quality of life. The data were collected at pre and post 7 day trial. Asthma control, health care utilization, and quality of life were reassessed at a 3 month follow-up after the trial for the asthma group. The results of our validity testing are beyond the scope of this paper.

#### Acceptance Evaluation

We assessed user acceptance using a brief survey and in-depth interviews with the participants. The user acceptance data were collected at the completion of the 7 day trial period from the asthma group participants only, because they represented the future users of the device. For the quantitative data, we devised a 7 item user acceptance survey to assess ADAM’s usefulness, user-friendliness, convenience, and social acceptableness on a 5 point scale, from strongly agree (5) to strongly disagree (1). The study staff went over each item with the participants. Total scores of 28 or higher on the 7 item acceptance survey were considered an indication of the participants’ satisfactory acceptance. The cutoff point was predetermined, because it would identify those who responded favorably in all 7 items by choosing either “agree” or “strongly agree”. The items were reviewed individually, and the proportion of “agree” and “strongly agree” responses for each question was computed.

To obtain in-depth feedback and perspectives on the device, we conducted brief semistructured individual interviews (10-15 minutes) with the asthma teens (n=42). The interviews were audiotaped and transcribed verbatim for analysis. *ATLAS.ti* 7 was used for data storage and management, and the basic qualitative description method was used to derive simple descriptions of the data in order to best understand the contents [21]. We used conventional content analysis to analyze the data [22]. There were two researchers that analyzed the data independently using content analysis techniques, and codes were compared. Any discrepancies between their analyses were discussed and reconciled. The coding was guided by the purpose of the interview, which was to determine the acceptability and usefulness of the device. No preconceived categories or a priori codes were used for analysis.

### Phase 3: Post Hoc Validation Phase

#### Modification of the Algorithm and Device Features

Phase 2 revealed the unstable performance of the device in detecting symptoms, particularly wheezing. On several occasions, we noted incremental wheezing counts even in a quiet environment. Moreover, during exit interviews, several of the participants reported incidents that suggested inaccuracies of the device (eg, “I saw the symptom number going up even when I wasn’t wheezing or coughing”). These indications of the suboptimal performance of the device prompted us to further refine the symptom algorithm to improve the sensitivity and specificity of it. Given the subtlety and wide variation of acoustic signatures of wheezes within and across individuals, the study team agreed that developing a stable and universal wheeze algorithm would be unattainable. We therefore decided to concentrate our focus on refining the cough algorithm. We enhanced the algorithm by incorporating a standard framework used in speech recognition to effectively filter in symptom sounds from speech and background noise. We manipulated the device so that it could record raw symptom sounds. In doing so, we could confirm whether the sounds identified by the algorithm were indeed asthma symptoms (true positive). To adequately assess specificity (true negative), the device automatically retained samples of the raw audio data that it captured. The device processed individual segments of 6 seconds. Whenever the algorithm identified a cough, the corresponding 6 seconds of raw audio samples were retained. Also, 6 second intervals of raw audio data were randomly retained when no coughs were identified. These audio files were retrieved, and allowed us to compute the performance metrics of the detection algorithm (ie, we were able to compare the device symptom counts against the audio data by manual listening). In Phase 2, activity levels were monitored inconsistently, resulting in incomplete data. To enable the built in accelerometer to monitor activity levels continuously for an extended time, we encouraged the participants to leave the device on all the time and to charge the battery whenever possible, in addition to using an external battery pack.

In the exit interview, some of the participants suggested that symptom and activity data be displayed in a graphical manner instead of raw numbers. We then modified the user interface so that symptom data would be presented in a bar graph that showed the aggregated number of coughs each hour along with the corresponding line chart of averaged activity scores (see the image on the right in Figure 1). In doing so, the users can readily keep track of changes in symptom patterns in the 24 hours, thus making the device data more meaningful. To enhance clinical relevance, we added a function that allowed the users to enter medication usage in the device, control and rescue medication separately, and when the medication was taken. This function was not only to assess the users’ medication adherence, but also to help the users visualize short- and long-term changes in symptoms after medication use. The users who understand how medications affect their symptoms might be more likely to adhere to their medication regimen.

#### Beta-Testing of the Enhanced Device

The small scale post hoc phase (Phase 3), involving ten adolescents with active symptoms, was designed to evaluate the adequacy of the enhanced algorithm, and to test the adequate operation of the revised and additional device features, including displaying data in a graphic form and recording medication use. The eligibility criteria and recruitment strategies were the same as those in Phase 1. The participants used the enhanced device for three consecutive days.

## Results

### Phase 1: Sample Demographic Characteristics and Symptom Algorithms

#### Summary

The demographic characteristics of the sample are summarized in Table 1.

**Table 1:** The demographic characteristics of the sample in Phase 1 (n=37).

Characteristics		
**Sex, n (%)**		
	Males	21 (57)
	Females	16 (43)
**Age**		
	Mean (SD)	14.7 years (1.4)
**Race, n (%)**		
	White	16 (43)
	Nonwhite	21 (57)
**Annual household income, n (%)**		
	Less than US $30,000	22 (59)
	$30,000 to 69,999	6 (16)
	$70,000 or more	7 (19)
	Missing	2 (5)

#### Asthma Symptom Severity

Of the sample of the participants (n=29) who provided raw symptom sounds, only 28% (8/29) reported their asthma was well controlled. Specifically, 14 teens reported nighttime symptoms once per week or more often, and 72% (21/29) had >2 days/week or more frequent daytime symptoms. About 80% (23/29) used rescue medication >2 days/week or more frequently, and all but one participant reported limited activities of some degree.

#### Development of Symptom Algorithms

**Figure 5:**
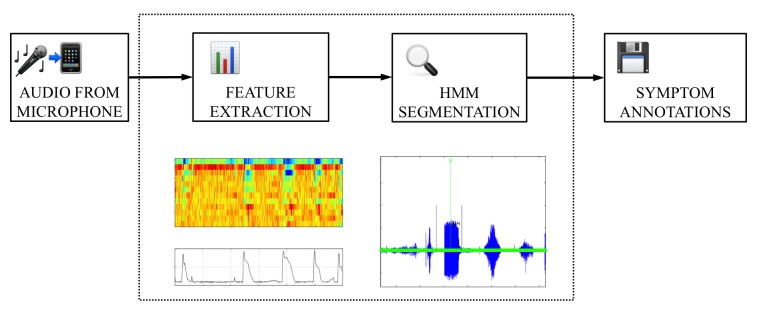
Schematized flow of the data processing used in the automated device for asthma monitoring (ADAM) application. HMM=hidden Markov model.

The algorithm for detecting coughs processed audio data in two steps following a standard pattern common to speech and other recognition frameworks. In the initial preprocessing step, the incoming stream of digital audio samples was arranged into fixed length frames from which a set of audio features (feature vector) was computed. The sequence of feature vectors was then passed to a hidden Markov model (HMM) Viterbi decoder (using a token passing implementation) [23,24], using HMMs trained on our database of symptom and background noises. This basic methodology has previously been shown to be applicable to the ambulatory detection of coughing sounds [25]. Subsequently, the number and times of the detected symptoms were stored in the platform device memory, and displayed for patient review. Figure 5 illustrates the schematic overview of the audio data processing by ADAM. Although our initial intention was to detect both coughing and wheezing, we were only able to successfully apply our automated techniques to coughs. The detailed technical descriptions about the development and validity of the symptom algorithm for coughs are reported elsewhere. The samples of wheezes gathered were too sparse, and varied too much across each individual teen, thus, we did not have enough data to reliably train an HMM for wheeze detection. The sensitivity (true positive) of the updated cough algorithm was 70% (21/30), and, on average, 2 coughs per hour were identified as false positive.

**Figure 6:**
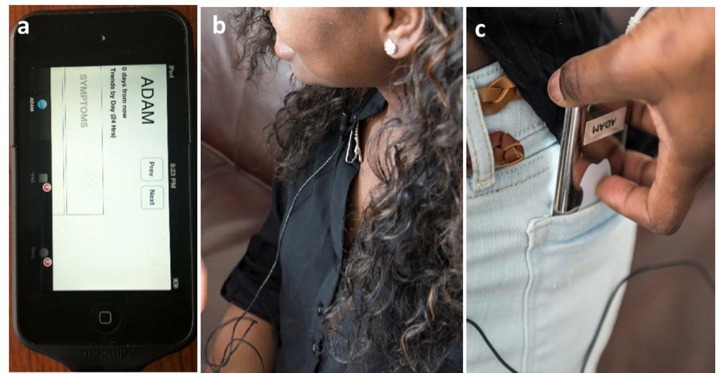
Images of the automated device for asthma monitoring (ADAM) with an external battery attached (a), and a teen wearing the device (b and c).

ADAM was also designed to measure activity levels using an incorporated accelerometer. However, we encountered challenges in monitoring activities consistently due to the device’s limited battery life. Later in our trial, we attached an external battery pack to the device to address the issue. Figure 6 shows the images of the prototype device with an external battery (a), and a teen wearing the device (b and c).

### Phase 2: Participants and Procedures

#### Recruitment and Procedures

A total of 84 teens (42 asthma teens and 42 nonasthma teens) participated in Phase 2. The sample characteristics are summarized in Table 2.

Both of the groups of teens, asthma and nonashthma, were recruited from the researcher-affiliated academic medical center. There was 73% (61/84) of the final sample that were recruited from the pediatric emergency department, some were from outpatient clinics (4%, 3/84), and some from recruitment fliers (23%, 19/84). All of the participants but one in the nonasthma group completed the 7 day trial. We learned that some schools do not allow students to use mobile devices during instructional hours. So, we provided an official letter addressed to a school administrator asking permission for the participants to continuously use the device during school hours. No participants reported any other issues related to using the device at school. All of the devices except for one were safely recovered. The missing device was assigned to a nonasthma teen, who did not complete the follow-up after enrollment.

**Table 2:** The demographic characteristics of the asthma and nonasthma groups in Phase 2 (N=84).

Characteristics		Asthma group	Nonasthma group
**Gender, n (%)**			
	Male	17 (40.5)	16 (38)
	Female	25 (59.5)	26 (62)
**Age**			
	Mean (SD)	15.2 years (1.5)	14.8 years (1.3)
**Race, n (%)**			
	White	16 (38)	29 (69)
	Nonwhite	24 (57)	13 (31)
	Missing	2 (5)	0
**Annual household income, n (%)**			
	Less than US $30,000	22 (52.4)	12 (28.6)
	US $30,000 to 69,999	7 (16.7)	11 (26.2)
	US $70,000 or more	12 (28.6)	19 (45.2)
	Missing	1 (2.3)	0

#### Results of the Acceptance Survey

Table 3 summarizes the responses for each acceptance item. The user acceptance data were collected only from the asthma group (n=42). Overall, the majority of the teens responded positively to each item. Particularly, 83% (35/42) of the asthma group reported having no trouble in remembering to wear the device for 24 hours to monitor their symptoms. Cronbach alpha of the 7 item acceptance survey was .78. The total acceptance scores ranged from 18 to 35 (mean 27.1, SD 4.4). Over 57% (24/42) of the sample responded with a total score of 28 and greater, indicating overall satisfaction with the device. No significant differences in acceptance were found by gender, age, race, family income, or the current status of symptom control (symptomatic vs asymptomatic). We computed correlations between the 7 acceptability items and the 5 items of the asthma control test. Significant negative relationships were found between the “embarrassment” item, the frequency of daytime symptoms (*r*=-.43, *P*=.004), and rescue medication use (*r*=-.45, *P*=.003). That is, those who reported higher levels of daytime symptoms or rescue medication use were more likely to be embarrassed about using the device in the presence of others.

**Table 3:** Summary of the user acceptance survey for each item (n=42).

Items	Agree, n (%)	Neutral, n (%)	Disagree, n (%)	Missing, n (%)
I like the idea of having the device record my asthma symptoms to monitor how well my asthma is being controlled and managed.	34 (81)	8 (19)	0	-
The device helped me be more aware of my asthma condition.	24 (57)	16 (38)	2 (5)	-
The device would make it easier for me to manage my asthma.	20 (47)	13 (31)	9 (21)	-
I don’t mind using the device for a week or longer for continuous monitoring of my asthma.	29 (69)	7 (17)	6 (14)	-
I found it easier operating the device than my peak flow meter.	23 (55)	9 (21)	2 (5)	8 (19)
I would not have any trouble remembering to wear it every morning throughout the day and put it by my bed for nighttime symptoms.	35 (83)	2 (5)	5 (12)	-
I would not be embarrassed to use the device in front of my friends.	30 (72)	3 (7)	9 (21)	-

#### Results of the Qualitative Interview Data

##### Categories of Acceptability

After establishing a preliminary set of codes after content analysis, two researchers presented them to other team members for their input and feedback. Revisions were made to the codes, and all of the interviews were recoded until a consensus was reached. The team then clustered the codes into categories that best described the acceptability of the device. We chose to create a series of descriptive data categories, as it fit the direct and exploratory nature of the work [26]. The initial set of categories was presented to the team. We created a final set of categories following further discussion and consensus with the team. Member checking was done by presenting the categories and codes to a group of adolescents with asthma (n=6) who did not participate in the interviews. The following are five categories of acceptability identified from the interview data, with examples of statements from the teens.

##### Comfortable and Cool

This category describes the cognitive and emotional aspect of using the device. The adolescents reported feeling comfortable wearing the device. They did not feel self conscious or embarrassed about using it around their peers, and they perceived the iPod format to be “cool” and “stylish”.

It was kinda’ cool the way y’all set it up, the iPod. It didn’t make it look like something suspicious. It made it look, you know, a regular person having an iPod.

Judy

I just kind of went through my regular activities, woke up, put the iPod in my pocket, went to school and told my friends...they were like, “dude, that’s awesome”...

Mark

##### Easy and Convenient

This category represents the participants’ perceptions about the levels of needed skills and convenience in using the device. The majority (n=37) described the device as being “easy” to use and understand. They concurred that the device was easy to operate and convenient to use daily.

Like, I could understand everything and it’s not like its difficult or inconvenient or anything. It just listens and you have to remember to keep it on you.

Jeremy

It was easy. Just put it in my pocket. Go to school and when I go to sleep, just put it by my bed. Simple. Then you wake up again and do the same thing all over.

Johnny

##### Useful and Helpful

The adolescents recognized that the device was useful and helpful in changing their perspectives on their asthma management, as well as conversing with others about their asthma. It assisted in the recognition of symptoms and triggers, and promoted the reflection of symptom-triggering behaviors and environments. Some of the participants stated that this awareness led to changes in their asthma self-management behaviors.

I really think it was a great experience because it really showed me during my activities, daily lives, and what I do, whether I am controlling it or what I shouldn’t be doing. If I’m around smoke or running, it shows me whether I’m coughing or wheezing or how good, how level my asthma is...

Emma

[I]t really like put it in front of me and I’m like “wow, I did not know that that was how bad I was today and yesterday I was worse” and it definitely helped me.

Alice

The numbers on the diary thing...helps with the triggers because if you’re around certain things or you’re doing certain activities and your numbers go up for coughing or wheezing, then you know that activity makes you cough or wheeze a lot...it really shows people...so if you think running probably affects your asthma that could really tell you instead of telling yourself, “oh I’m out of breath. I’m wheezing.” That tells you how many wheezes...it shows you whether or not are you controlling your asthma or is it out of control...

Tom

The participants also recognized that using the device was helpful in initiating and stimulating dialogues with peers, family, and health professionals about asthma. While this often started as a conversation about the device itself, it also led to deeper conversations about their health condition with others.

A lot of people question me. Like what the device was...And I kind of explained to them that I was doing as asthma study and they kind of took it, “Oh that pretty cool”, but others “What is that? I never knew you had asthma.” So, I had to explain to them...how the entire device worked.

Vicky

My friends are pretty much alright, so they say “Oh I think that’s pretty cool” or a couple of my friends were like “Oh I wish they had stuff like that for me. Where did you find out from?” Because I have a couple of friends who have asthma too and um one of my friends who doesn’t have asthma, she was really, really like really excited. “Maybe they’ll figure out something to help manage your asthma.” I’m like I hope so...

Justin

##### We Would Wear It

Most of the participants (n=37) concurred that the device was conducive to everyday use for anyone who has asthma, and would recommend it to other teens with asthma.

[I would recommend it] definitely if they’re struggling with asthma, I feel like it would be a really good, easy way to see how you’re actually doing.

Mickey

##### Suggestions for Improvement

The participants made several constructive suggestions for improving the device. Although the majority of the teens (n=23) favored using an iPod as a platform, 19 participants stated that they would prefer a smaller and lighter device. Additionally, 18 participants recommended simplified accessories (microphone, cords) that would make it easier and more convenient to use. There were six of the adolescents that suggested making the device available in a smartphone application (app) format, and three suggested that the device be equipped with audio prompts for worsening symptoms or reminders. For some, the numeric and graphic presentation of the symptoms displayed on the device screen were confusing, suggesting the need for further refinement of the user interface of the device.

### Phase 3: Sample and Enhanced Device Data Summary

Of the 10 teen participants in Phase 3, six were females (60%), and seven were black or African Americans (70%), and from families of an annual income under US $30,000. Of a total of 146,167 data points recorded in the device (mean 18,271 data/teen), coughs were recorded 3784 times (2.59%). The device sampled 27,404 sound clips (6 seconds each). The total number of accelerometer data of one minute length each was 36,040 (4004 per teen). The volume of data indicated that activity levels were monitored for, on average, 2.7 days per participant, accounting for most of the 3 day period. The range of the accelerometer data was 0-24 (mean 0.83, SD 2.89). There was significant correlation found between the number of coughs and the accelerometer data (*r*=.037, *P*<.001), indicating that the greater number of coughs was associated with the higher activity levels. The medication tracking feature was used by eight teens (80%, 8/10) for 3 days. On average, each teen reported using controller medications three times and rescue medication four times throughout the three day period. All of the participants concurred that the graphical presentations of the symptom and activity data helped their understanding of it, and the medication tracking feature was easy to use.

## Discussion

### Asthma Symptom Monitoring

Accurate symptom monitoring by patients is the most fundamental antecedent to effective asthma management, yet existing monitoring strategies have not been conducive to adolescents’ cooperation, or yielded accurate or clinically useful information [13,18-20]. Having recognized these limitations, we attempted to offer an innovative alternative to symptom monitoring through the development of a new device, ADAM, that would stimulate an adolescents’ engagement, and ultimately lead to effective asthma management. Developing and validating a new device is complex, inherently involving multiple steps of meticulous planning and implementation, as demonstrated in this study. The objective of this paper was to describe the procedural elements that had been chosen to develop and validate the ADAM prototype to quantify asthma symptoms, and to examine the user acceptance of the device. A few attempts have been made to quantify the symptoms such as coughs and wheezes. However, most of these attempts have not been translated into addressing clinical issues, such as the inadequate patient monitoring of the symptoms. To our knowledge, this was the first attempt to incorporate symptom quantifying technology into mobile technology in developing a device that facilitates continuous monitoring of symptoms in vivo, particularly in adolescents with asthma. Because of the fluctuating nature of asthma symptoms during a given day (eg, more symptoms during nighttime than daytime), researchers have advocated for continuous symptom monitoring to obtain the abundance of useful information that it can generate to help health care providers make informed decisions regarding treatment [27].

ADAM was intended to automatically monitor coughs, wheezes, and activity level continuously for 24 hours. We sampled and validated the sounds of wheezes and coughs by employing a rigorous and lengthy process, and delineated algorithms of these symptoms based on the sound samples. Unlike coughs that were rather conspicuous, wheezes were subtle, adventitious, and had wide variations in acoustic manifestations, which made it difficult to establish a sensitive, generalizable algorithm that could sufficiently distinguish the wheezes from other noises. While wheezing sounds were often captured using a stethoscope and a quiet laboratory setting in earlier studies [28-33], our wheeze algorithm was based on the sound data captured using an ordinary external microphone in everyday environments that were exposed to a wide range of noises. In the future, advanced sound capturing technology could help delineate wheeze algorithms effectively, while ruling out other noises. Due to the challenges involved in distinguishing wheezes from environmental noises using regular microphones, we decided to concentrate our efforts on coughs alone for the further refinement of the device. A cough is the most common symptom experienced by pediatric patients with uncontrolled asthma [34-37]. Therefore, we believe detecting coughs alone could add value to this device as a monitoring tool. The algorithm of coughs underwent several modifications throughout the study period to improve its performance. Beta-testing of the device raised concerns about accuracy, as it had initial challenges in differentiating noises (eg, door slamming, some speech sounds) from legitimate coughs. As a result, the algorithm was further refined and strengthened through multiple iterations. The sensitivity (true positive) of the updated algorithm was 70% (21/30), and, on average, 2 coughs per hour were identified as false positive. Further refinement of the algorithm is warranted to increase the accuracy and overall performance.

We used an existing mobile phone, the iPod, as a platform for processing, analyzing, and storing the data transmitted from a microphone. The iPod was selected as an age appropriate platform for ADAM because of its widespread ownership among, and potential appeal to, adolescents. The popular nature of the iPod among teens could make the device inconspicuous, should it be used for symptom monitoring in social settings. Peer influence is one of the barriers to self-management in adolescents [38]. In fact, adolescents often feel uncomfortable or embarrassed about their asthma, and are reluctant to take their asthma medication in the presence of their friends [39-44]. Therefore, using an inconspicuous platform such as the iPod was essential for the device to be accepted and utilized by adolescent users as intended. In addition, the iPod operating system (iOS) has a built in accelerometer that keeps track of the intensity of the users’ movement, which indicates the level of activity. This function was incorporated in ADAM not only to monitor any impairment in daily activities, but also to facilitate the understanding of the symptoms in the context of the users’ activity levels. That is, the device could potentially help to make distinctions between the symptoms that occur in connection with activity (eg, exercise induced symptoms), and those occurring independent of activities. Because the device design enables continuous monitoring of the symptoms and activities in real life situations, the new device provides a powerful option for comprehensive asthma monitoring that is also developmentally compelling.

### Limitations

Several limitations of the study are noteworthy. First, our attempt to develop a reliable algorithm of wheezes was faced with challenges because of the wide variations of the acoustic signature of wheezing. Furthermore, the subtle nature of wheezing presented difficulties in detecting the sound using an external microphone in the teens’ everyday life contexts. Therefore, wheezing detection by the device was not accomplished. Second, we learned later in Phase 2 that in order for the iPod’s built in accelerometer function to run continuously, the device should not to be on a “sleep” mode, which depleted battery power rapidly. As a result, we were unable to collect sufficient activity data to correlate with the number of symptoms. To remedy the issue, we attached an external battery pack to the iPod, which made the device bulky and less attractive, as commented by some of the teens. Third, some of the teens felt the cord of the external microphone was a nuisance. Further incorporation of advanced technology in the modification of the device (eg, power efficient operation of accelerometer, wireless microphone) is necessary to overcome the identified technical challenges and increase user convenience. Fourth, the user acceptance was based on only 7 days’ use of the device. Therefore, we were unable to make any predictions about changes in the users’ perceptions and attitudes toward the device over time. Further research is needed to examine the teens’ attitudes over time, and how the attitudes affect the sustainability of the device. Last, although we speculated that displaying symptom data in a graphical form, a modification done in Phase 3, would aid the users’ grasp of the clinical meaning of the detected symptoms, we did not collect data to support the assumption. Likewise, it is yet to be determined whether the medication tracking function we added in Phase 3 would improve medication adherence. Since Phase 3, we have further updated the user interface to make the device more user-friendly and effective in communicating data with the users (eg, automated “pop-up” messages triggered by the levels of detected symptoms). In addition, as suggested by some of the teens, the ADAM system is being developed as an “app” downloadable to mobile iOS systems, which will further enhance the device’s accessibility in the future.

### Positive Reviews

Despite the identified limitations, the new device was positively reviewed and accepted by the adolescent users who found it appealing, convenient, and conducive to daily use. The identified potential benefits of the device shed light on the positive impact of the device on promoting self-management, and ameliorating the burdens of asthma in adolescents. The endorsement of the device from the adolescent users is an encouraging first step to adherence to adequate self-monitoring, which has been challenging and elusive, particularly in this age group. Incorporating suggestions from the users in enhancing the device is critical to ensure its consistent use, and to increase its developmental and clinical relevance.

### Conclusions

In summary, this new device can potentially automate daily symptom monitoring with minimal intrusiveness and maximum accuracy. Due to its foreseen safety, noninvasiveness, objectivity, convenience, user-friendliness, and cost containment, the approach has the potential to greatly enhance asthma management by adolescents and health care providers, thereby reducing the risk of inappropriate treatment and ameliorating asthma related impairments. As suggested in our qualitative data, this device has the potential to bring about changes in patient behaviors, such as avoiding triggers and adjusting medications by increasing the patients’ awareness through real time assessment of symptoms and feedback. Further study is needed to evaluate the efficacy of the device in improving self-management and asthma outcomes.

## Abbreviations

ADAM: automated device for asthma monitoring
app: application
HMM: hidden Markov model
iOS: iPod operating system
PEF: peak expiratory flow
teens: teenagers

## References

[1] Akinbami LJ, Moorman JE, Liu X (2011). Asthma prevalence, health care use, and mortality: United States, 2005-2009. Natl Health Stat Report.

[2] Center for Disease Control and Prevention CDC.

[3] Center for Disease Control and Prevention Center for Disease Control and Prevention.

[4] Akinbami LJ, Moorman JE, Garbe PL, Sondik EJ (2009). Status of childhood asthma in the United States, 1980-2007. Pediatrics.

[5] Bloom B, Cohen RA, Freeman G (2011). Summary health statistics for U.S. children: National health interview survey, 2010. Vital Health Stat 10.

[6] Calmes D, Leake BD, Carlisle DM (1998). Adverse asthma outcomes among children hospitalized with asthma in California. Pediatrics.

[7] Akinbami LJ, Schoendorf KC (2002). Trends in childhood asthma: Prevalence, health care utilization, and mortality. Pediatrics.

[8] Gibson PG, Ram FS, Powell H (2003). Asthma education. Respir Med.

[9] Warsi A, Wang PS, LaValley MP, Avorn J, Solomon DH (2004). Self-management education programs in chronic disease: A systematic review and methodological critique of the literature. Arch Intern Med.

[10] Wolf FM, Guevara JP, Grum CM, Clark NM, Cates CJ (2003). Educational interventions for asthma in children. Cochrane Database Syst Rev.

[11] Guevara JP, Wolf FM, Grum CM, Clark NM (2003). Effects of educational interventions for self management of asthma in children and adolescents: Systematic review and meta-analysis. BMJ.

[12] Bruzzese JM, Bonner S, Vincent EJ, Sheares BJ, Mellins RB, Levison MJ, Wiesemann S, Du Y, Zimmerman BJ, Evans D (2004). Asthma education: The adolescent experience. Patient Educ Couns.

[13] Bhogal S, Zemek R, Ducharme FM (2006). Written action plans for asthma in children. Cochrane Database Syst Rev.

[14] National Heart, Lung, and Blood Institute US Department of Health and Human Services.

[15] Sarver N, Murphy K (2009). Management of asthma: New approaches to establishing control. J Am Acad Nurse Pract.

[16] Horak E, Grässl G, Skladal D, Ulmer H (2003). Lung function and symptom perception in children with asthma and their parents. Pediatr Pulmonol.

[17] Halterman JS, Yoos HL, Kitzman H, Anson E, Sidora-Arcoleo K, McMullen A (2006). Symptom reporting in childhood asthma: A comparison of assessment methods. Arch Dis Child.

[18] Adams RJ, Boath K, Homan S, Campbell DA, Ruffin RE (2001). A randomized trial of peak-flow and symptom-based action plans in adults with moderate-to-severe asthma. Respirology.

[19] Yoos HL, Kitzman H, McMullen A, Henderson C, Sidora K (2002). Symptom monitoring in childhood asthma: A randomized clinical trial comparing peak expiratory flow rate with symptom monitoring. Ann Allergy Asthma Immunol.

[20] Wensley D, Silverman M (2004). Peak flow monitoring for guided self-management in childhood asthma: A randomized controlled trial. Am J Respir Crit Care Med.

[21] Sandelowski M (2000). Whatever happened to qualitative description?. Res Nurs Health.

[22] Hsieh HF, Shannon SE (2005). Three approaches to qualitative content analysis. Qual Health Res.

[23] Young S, Evermann G, Gales M, Hain T, Kershaw D, Liu XA, Moore G, Odell J, Ollason D, Povey D, Valtchev V, Woodland P (2006). The HTK book (for HTK version 3.4).

[24] Young SJ, Russell NH, Thornton JHS (1989). Token passing: A simple conceptual model for connected speech recognition systems.

[25] Matos S, Birring SS, Pavord ID, Evans DH (2007). An automated system for 24-h monitoring of cough frequency: The Leicester cough monitor. IEEE Trans Biomed Eng.

[26] Kearney MH (2001). Levels and applications of qualitative research evidence. Res Nurs Health.

[27] Rietveld S, Oud M, Rijssenbeek-Nouwens LH, Vaghi D, Dooijes EH (1999). Characteristics and diagnostic significance of spontaneous wheezing in children with asthma: Results of continuous in vivo sound recording. J Asthma.

[28] Fiz JA, Jané R, Izquierdo J, Homs A, García MA, Gomez R, Monso E, Morera J (2006). Analysis of forced wheezes in asthma patients. Respiration.

[29] Beck R, Dickson U, Montgomery MD, Mitchell I (1992). Histamine challenge in young children using computerized lung sounds analysis. Chest.

[30] Rietveld S, Kolk AM, Prins PJ, Colland VT (1997). The influence of respiratory sounds on breathlessness in children with asthma: A symptom-perception approach. Health Psychol.

[31] Baughman RP, Loudon RG (1984). Quantitation of wheezing in acute asthma. Chest.

[32] Rietveld S, Oud M, Dooijes EH (1999). Classification of asthmatic breath sounds: Preliminary results of the classifying capacity of human examiners versus artificial neural networks. Comput Biomed Res.

[33] Malmberg LP, Sovijärvi AR, Paajanen E, Piirilä P, Haahtela T, Katila T (1994). Changes in frequency spectra of breath sounds during histamine challenge test in adult asthmatics and healthy control subjects. Chest.

[34] Rietveld S, Rijssenbeek-Nouwens LH (1998). Diagnostics of spontaneous cough in childhood asthma: Results of continuous tracheal sound recording in the homes of children. Chest.

[35] Wildhaber J, Carroll WD, Brand PL (2012). Global impact of asthma on children and adolescents' daily lives: The room to breathe survey. Pediatr Pulmonol.

[36] Davis KJ, Disantostefano R, Peden DB (2011). Is Johnny wheezing? Parent-child agreement in the childhood asthma in America survey. Pediatr Allergy Immunol.

[37] Price D, Ryan D, Pearce N, Bawden R, Freeman D, Thomas M, Robson L (2002). Prim Care Respir J.

[38] Rhee H, Belyea MJ, Ciurzynski S, Brasch J (2009). Barriers to asthma self-management in adolescents: Relationships to psychosocial factors. Pediatr Pulmonol.

[39] Cohen R, Franco K, Motlow F, Reznik M, Ozuah PO (2003). Perceptions and attitudes of adolescents with asthma. J Asthma.

[40] Kyngas H, Rissanen M (2001). Support as a crucial predictor of good compliance of adolescents with a chronic disease. J Clin Nurs.

[41] Rhee H, Wenzel J, Steeves RH (2007). Adolescents' psychosocial experiences living with asthma: A focus group study. J Pediatr Health Care.

[42] Ayala GX, Miller D, Zagami E, Riddle C, Willis S, King D (2006). Asthma in middle schools: What students have to say about their asthma. J Sch Health.

[43] La Greca AM, Bearman KJ, Moore H (2002). Peer relations of youth with pediatric conditions and health risks: Promoting social support and healthy lifestyles. J Dev Behav Pediatr.

[44] Mulvaney SA, Ho YX, Cala CM, Chen Q, Nian H, Patterson BL, Johnson KB (2013). Assessing adolescent asthma symptoms and adherence using mobile phones. J Med Internet Res.

